# Vulvovaginal Candidiasis in Pregnancy—Between Sensitivity and Resistance to Antimycotics

**DOI:** 10.3390/jox13030023

**Published:** 2023-07-05

**Authors:** Nicoleta-Maricica Maftei, Manuela Arbune, Costinela Valerica Georgescu, Alina Mihaela Elisei, Alina Viorica Iancu, Alin Laurentiu Tatu

**Affiliations:** 1 Faculty of Medicine and Pharmacy, Research Centre in the Medical-Pharmaceutical Field, University “Dunărea de Jos”, 800010 Galati, Romania; costinelag@gmail.com (C.V.G.);; 2 Medical Laboratory Department, “Sfântul Ioan” Children’s Emergency Hospital, 800487 Galați, Romania; 3 Clinical Medical Department, Faculty of Medicine and Pharmacy, University “Dunărea de Jos”, 800010 Galati, Romania; manuela.arbune@ugal.ro (M.A.); dralin_tatu@yahoo.com (A.L.T.); 4 Infectious Diseases Department, Clinical Hospital of Infectious Diseases “Sf. Cuvioasa Parascheva”, 800179 Galati, Romania; 5 Department of Public Health, Clinical Hospital of Obstetrics and Gynecology “Buna Vestire“, 800151 Galati, Romania; 6 Medical Laboratory Department, Clinical Hospital of Infectious Diseases “Sf. Cuvioasa Parascheva”, 800179 Galati, Romania; 7 Department of Morphological and Functional Sciences, Faculty of Medicine and Pharmacy, University “Dunărea de Jos”, 800010 Galati, Romania; 8 Research Center in the Field of Medical and Pharmaceutical Sciences, ReFORM-UDJ, 800010 Galati, Romania; 9 Dermatology Department, Clinical Hospital of Infectious Diseases “Sf. Cuvioasa Parascheva”, 800179 Galati, Romania

**Keywords:** pregnancy, antifungal, candidiasis, fungal resistance

## Abstract

Vulvovaginitis with *Candida* spp. is the most common infection in women and the rate is increased during pregnancy. Antifungal prescription in pregnant women continues to present challenges and the decision must balance the risk of fetal toxicity with the benefits to the fetus and mother. Starting from the idea that clotrimazole is the most recommended antifungal in candidal vaginitis in pregnancy, we tested the sensitivity of different species of *Candida* spp. to other azoles, polyenes, and antimetabolites. This retrospective study (January to June 2019) assessed 663 pregnant women hospitalized for various pregnancy-related symptoms in which samples of phage secretion were taken. The laboratory results confirmed 21% of cases, indicating 140 positive mycologic samples. In this study, vaginal candidiasis was mostly related to the first trimester of pregnancy (53.57%,) and less related in the last trimester (17.14%). *Candida albicans* was the most frequent isolated strain in this study, accounting for 118 cases, followed by 16 strains of *Candida glabrata* and 6 cases of *Candida krusei*. The highest sensitivity for *C. albicans* was found in azoles, mostly in miconazole (93.2%), while *C. krusei* was completely resistant to polyene with low sensitivity in antimetabolites and even in some azoles, such as fluconazole. In our study, higher resistance rates to flucytosine were found, with *C. glabrata* and *C. krusei* exhibiting greater resistance than *C. albicans*.

## 1. Introduction

Pregnancy enhances vulnerability to infections, although it is considered to be a physiological state, and increases the rate of spontaneous abortions, congenital malformations, fetal toxicity, and premature births. *Candida* spp. are the most common fungal infections during pregnancy, with an increased rate of 30% compared with 20% in nonpregnant women [1,2].

Most of the infections are recorded in the first trimester of pregnancy, which brings forward the risk of the occurrence of antimycotic teratogenic effects and imposes the usage of minimum effective doses of antimycotics during this key fetal development period [3].

New antifungal drugs have been developed in the last decade. However, there is a lack of complete clarity in terms of the optimum antifungal regimens and doses during pregnancy, especially considering the changes in body composition and variations in maternal pharmacokinetics [4].

The major antifungal classes are azoles, polyenes, antimetabolites, and echinocandins, each with distinct acting mechanisms. According to the Food and Drugs Administration (FDA), these antifungal drugs are classified into five groups that are related to the risks in pregnancy (Table 1).

**Figure 1:**
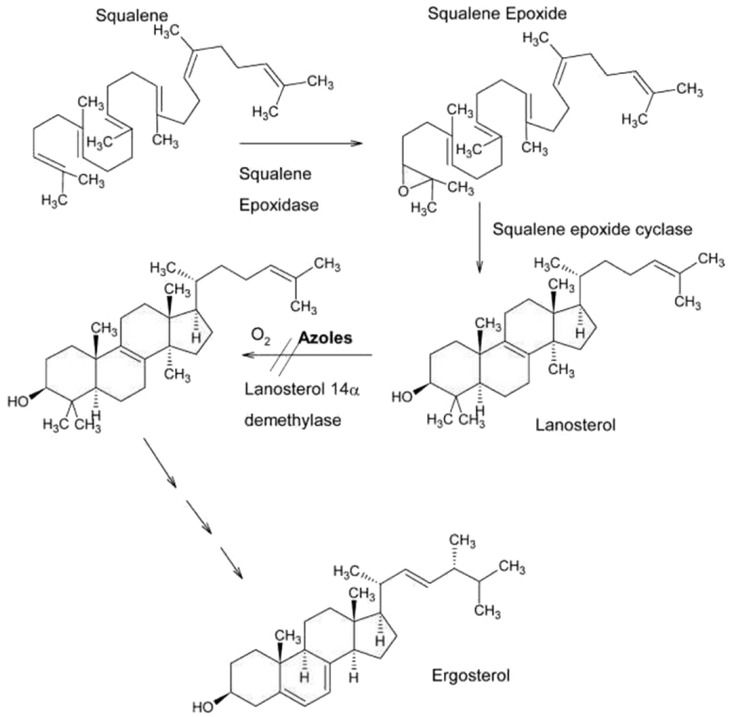
Azole mechanism of antifungal action. Source: https://www.pharmacologicalsciences.us/pharmaceutical-chemistry/mechanism-of-action.html (accessed on 12 February 2023).

Azole drugs alter the fungal membrane’s structure and permeability by blocking 14-demethylase activity and the selective inhibition of cytochrome P_450_-dependent lanosterol 14α-demethylase in the pathway of ergosterol synthesis. Ergosterol biosynthesis inhibitors such as the allylamines (naftifine, terbinafine) interfere with ergosterol synthesis by inhibiting the enzyme involved in the first ergosterol synthesis stages, namely squalene epoxidase (Figure 1).

This inhibition leads to the accumulation of large quantities of squalene and a reduction in the quantity of ergosterol, leading to permeability disorders, the fragmentation of parietal cell structures, and, in the end, the death of the fungal cell [6]. Ketoconazole is obtained by chemical synthesis with the active substance cis-1-acetyl-4/4-2-2,4-dichlorophenol-2-1-*H*-imidazole-1-yl-metyl-1,3-dioxolan-4yl-methoxy-nilpiperazine. Fluconazole contains two triazole structures (2-(2,4-difluorophenyl)-1,3-*bis*(1*H*-1,2,4-triazole-1-yl) propan-2-ol) and is the most used antifungal agent [6].

Polyene drugs, including amphotericin B and nystatin, bind to the cellular membrane, causing modifications to the fungal membrane’s structure and permeability through selective ergosterol binding. Adding sodium deoxycholate (D-AMB) to the amphotericin B formula allows for parenteral administration. The toxicity profile of polyenes has been improved by nanotechnology, as liposomal nystatin has been developed by being embedded into liposomes structures of dimyristoyl phosphatidylcholine and dimyristoyl phosphatidylglycerol [6].

Glucan synthesis inhibitors such as caspofungin, micafungin, and anidulafungin impair the synthesis of 1,3-beta-D-glucan, a substance that is required in the composition of the fungal cellular wall. Glucan fibers, together with chitin, are organized into a firm reticular structure that provides cellular shape and strength, both of which are parameters that are involved in essential cell functions such as osmosis, growth, and division [6].

Antimetabolites represented by small molecules like the DNA matrix cause the incorrect synthesis of DNA molecules (flucytosine). Chemically speaking, 5-flucytosine is a fluoridated pyrimidine. The action mechanism of this antifungal is different from the other antifungals, with the fungal cell being taken over and transformed by a deamination process into 5-fluorouracil, a compound that replaces the uracil within the fungal RNA. It also inhibits thymidylate synthase by 5 fluorodeoxyuridine monophosphate, an essential enzyme in RNA synthesis [6]. This study aimed at establishing the sensitivity and resistance of various *Candida* strains to antimycotics because clotrimazole is the most recommended antifungal in candidal vaginitis in pregnancy.

## 2. Materials and Methods

### 2.1. Sampling The Cases

From January to June of 2019, 663 pregnant women were admitted to the “Buna Vestire” Clinical Obstetrics-Gynaecology Hospital of Galati with a clinical presumptive diagnosis of genital candidiasis; however, following laboratory analyses, only 140 women were finally diagnosed with a clinical diagnosis of genital candidiasis. The laboratory results confirmed 21% of cases to have positive mycologic samples after being statistically analyzed. Sampling was conducted following the best practices outlined in standards and codes, and all protection measures for both patients and medical staff were complied with.

### 2.2. Laboratory Works

*Candida* strains were isolated from patients presenting clinical symptoms of vaginal infections. The samples were collected aseptically at the slit lamp. Cotton swabs (sterile) were gently smeared around the vaginal region and quickly transferred to Sabouraud dextrose broth with a pH of 5.6 that contained 4% dextrose and 1% peptone in distilled watercombined with 5.0 mg of chloramphenicol. The culture tubes thus prepared were incubated at 37 °C (24–48 h).

The reference standard strains were *C. albicans* and *C. parapsilopsis*, which were used to emphasize various strains. Microscopic examination was conducted using 10% potassium hydroxide (KOH) according to the method described in [7].

### 2.3. Mycologic Culture

Cultivation was undertaken on a chloramphenicol-free agar dextrose Sabouraud culture environment, which inhibits the multiplication of contaminated bacteria and allows for the good development of yeasts. On culture environments, medical yeasts develop within 48–72 h of incubation. The incubation temperature after seeding was 36–37 °C. Because the bacterial contamination might compromise the species identification stage, Gram-stained smears were made after obtaining the first culture, which were then examined with an inverted microscope. In order to obtain a pure culture, replication may be performed in order to eliminate other bacteria.

In order to determine the species and obtain the antifungigram, the Candifast kit was used. For testing, the antimycotics amphotericin and nystatin were used from the class of azoles, econazole, fluconazole, ketoconazole, miconazole, and polyenes, as well as flucytosine from the antimetabolites class.

### 2.4. Statistical Analysis

The sensitivity and resistance of various Candida strains to antifungal drugs was retrospectively analyzed by statistical methods using the statistical software SPSS 24.0. The Fisher test and the Relative Risk (RR) indicator were used with a confidence interval (IC95%), and we present the *p*-values generated by this test.

## 3. Results

Vulvovaginitis with *Candida* spp. is the most common infection in women and the rate is increased during pregnancy. Antifungal prescription in pregnant women continues to present challenges, and the decision must balance the risks of fetal toxicity with the benefits to fetus and mother.

**Figure 2:**
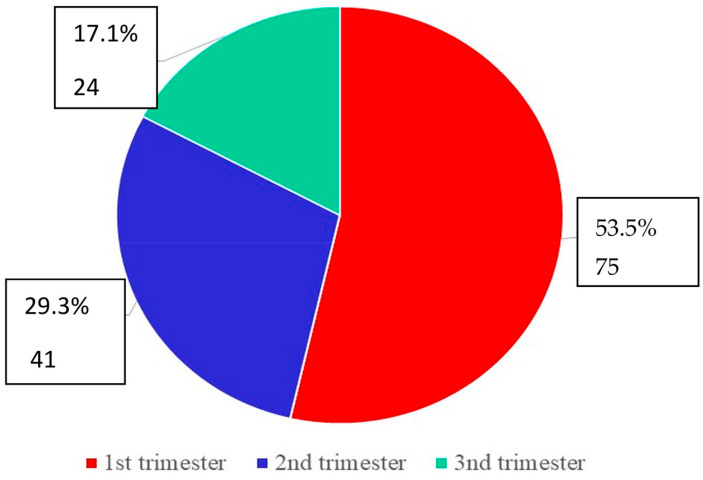
Distribution of vaginal candidiasis according to the stage of pregnancy.

In our study, vaginal candidiasis was mostly related to the first trimester of pregnancy (53.57%,) and was less common in the last trimester (17.14%) (Figure 2).

**Figure 3:**
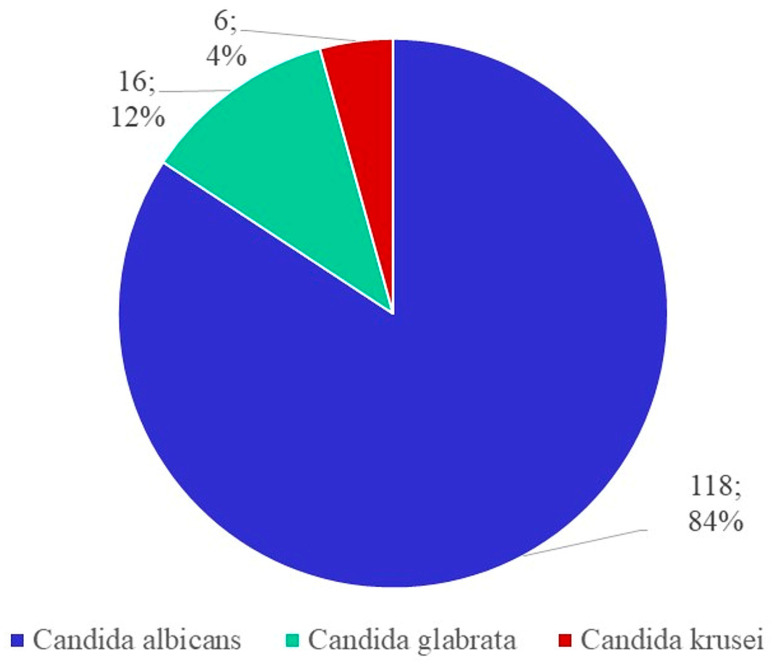
The frequency of different *Candida* spp.

*C. albicans* was the most frequent isolated strain in our study, accounting for 118 cases, followed by 16 strains of *C. glabrata* and 6 cases of *C. krusei* (Figure 3).

The highest sensitivity for *C. albicans* was found in azoles, mostly in miconazole (93.2%), while polyenes and antimetabolites showed much lower sensitivity (Table 2).

The estimated risk of *Candida albicans* was neither significantly higher in patients with polyene resistance (RR = 1.06; IC95%: 0.86–1.31; *p* = 0.691) nor a protection factor in those sensitive to polyenes (RR = 1.09; IC95%: 0.80–1.48; *p* = 0.691).

Compared with the azoles treatment, the resistance to amphotericin induced a significantly higher estimated risk of *Candida albicans*:

- Risk was nine times higher compared to miconazole resistance (RR = 9.0; IC95%: 4.54–17.18; *p* = 0.001);
- Risk was more than seven times higher compared with resistance to fluconazole (RR = 7.20; IC95%: 3.91–13.30; *p* = 0.001);
- Risk was four times higher compared with resistance to ketoconazole (RR = 4.0; IC95%: 2.55–6.27; *p* = 0.001);
- Risk was three times higher compared with resistance to econazole (RR = 3.0; IC95%: 2.04–4.41; *p* = 0.001).

**Figure 4:**
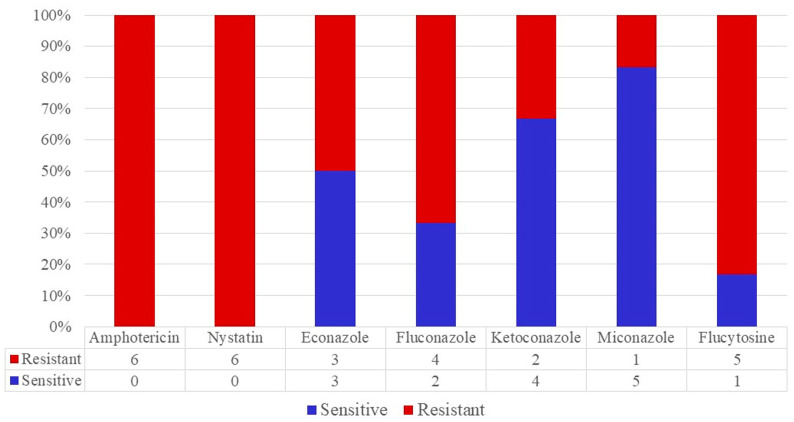
The antifungigram profile of *C. krusei* strains.

**Figure 5:**
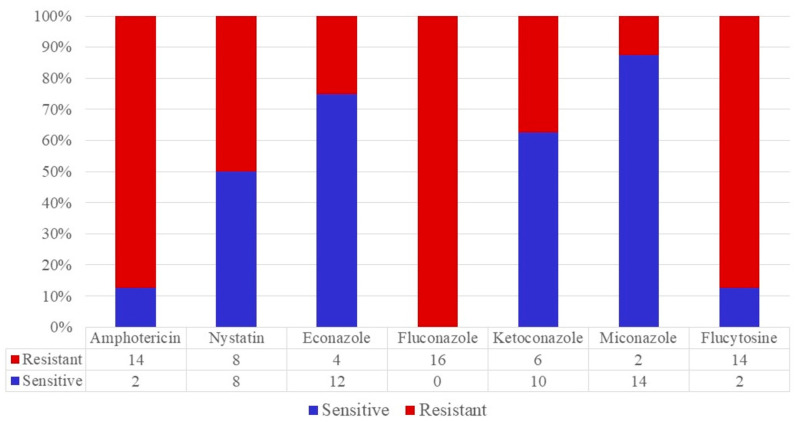
The antifungigram profile of *C. glabrata* strains.

*C. krusei* was completely resistant to polyene with low sensitivity in antimetabolites and even in some azoles, such as fluconazole. Although resistance to miconazole is higher than in *C. albicans*, this drug has the best potency based on sensitivity in over 80% of the strains, including *C. krusei (*Figure 4 and Figure 5*)*.

Compared with the azoles treatment, resistance to nystatin induced a significantly higher risk of infection with *Candida albicans*:

- Risk was 8.5 times higher compared with resistance to ketoconazole (RR = 8.50; IC95%: 4.28–16.90; *p* = 0.001);
- Risk was approximately seven times higher compared with the resistance to fluconazole (RR = 6.80; IC95%: 3.68–12.60; *p* = 0.001);
- Risk was 3.78 times higher compared with resistance to miconazole (RR = 3.78; IC95%: 2.40–5.94; *p* = 0.001);
- Risk was approximately three times higher compared with resistance to econazole (RR = 2.83; IC95%: 1.92–4.18; *p* = 0.001).

From the azoles, resistance to econazole induced a significantly higher risk towards miconazole (RR = 3.0; IC95%: 1.41–6.40; *p* = 0.004) or fluconazole (RR = 2.40; IC95%: 1.20–4.80; *p* = 0.016).

Sensitivity to azoles, compared with the sensitivity to polyenes (RR = 0.47; IC95%: 0.40–0.55; *p* = 0.001) or antimetabolites (RR = 0.28; IC95%: 0.20–0.38; *p* = 0.001), represents a protection factor in the positivation of *Candida albicans* strains.

Compared with resistance to azoles, the estimated risk of *Candida albicans* was significantly higher in patients resistant to antimetabolites (RR = 3.60; IC95%: 2.65–4.90; *p* = 0.001).

Compared with treatment with azoles, the resistance to polyenes (RR = 2.40; IC95%: 1.49–3.85; *p* = 0.003) or antimetabolites (RR = 2.10; IC95%: 1.26–3.49; *p* = 0.01) induced a two times higher estimated risk of *Candida glabrata*.

Sensitivity to azoles compared with sensitivity to polyenes (RR = 0.42; IC95%: 0.26–0.67; *p* = 0.003) or antimetabolites (RR = 0.48; IC95%: 0.29–0.79; *p* = 0.01) represents a protection factor in the positivation of the *Candida glabrata* strains.

Compared with the azoles treatment, resistance to antimetabolites induced a two times higher risk of *Candida krusei* (RR = 2.0; IC95%: 1.43–2.79; *p* = 0.004).

Sensitivity to azoles, compared with sensitivity to polyenes (RR = 0.63; IC95%: 0.44–0.91; *p* = 0.036) or antimetabolites (RR = 0.50; IC95%: 0.35–0.70; *p* = 0.004), represents a protection factor in the positivation of the *Candida krusei* strains.

In our study, higher resistance rates to flucytosine were found in *C. glabrata* and *C. krusei* than in *C. albicans.*

## 4. Discussion

The resistance to antifungal drugs is increasing. The choice of the right antifungal regiment for vaginal candidiasis in pregnant women must consider the general antibiotic stewardship principles, as well as drug sensitivity and resistance data related to fungal resistance tests. Nevertheless, these tests are time-consuming and waiting for results it is not always possible, requiring a first-line regimen based on local antifungal resistance data.

*Candida* spp. infection is often asymptomatic during pregnancy. Despite the absence of clinical symptoms, vaginal colonization has been associated with clinical events such as premature birth [8,9] and, in rare cases, intra-amniotic infection [10]. Numerous studies have described an association between maternal vaginal colonization and the subsequent colonization of the newborn [10,11]. *Candida* spp. represent a major risk factor for systemic infections in newborns and are associated with increased mortality [2,12]. Women who were colonized with *Candida* spp. in the second trimester of pregnancy had higher premature birth rates and lower fetal birth weight compared with colonization in the first trimester of pregnancy [13,14,15]. Thus, the antifungal prescription must consider the fetal risk of teratogenic effects and the usage of minimum effective doses [5].

Other conditions related to pharmacokinetics and pharmacodynamics during pregnancy, such as increased cardiac flow, intestinal blood flow, gastric pH, and serum albumin concentration, were considered for antifungal management. Furthermore, the changes in renal filtration and elimination, together with the changes in kidney receptor sensitivity, either increasing (2A6, 2C9, 2D6, 3A4) or decreasing (1A2, 2C19), highlight the kidney clearance of antifungal drugs [16,17,18].

Although clotrimazole is the most recommended antifungal in candidal vaginitis in pregnancy, miconazole is the preferred antifungal drug, whatever the species of vaginal candidiasis. Although miconazole is categorized in class C for pregnancy risk, the topical vaginal formulations used in clinical practice during the first trimester did not prove to have harmful effects [19].

Conversely, nystatin and amphotericin have the lowest sensitivity for all species and should not be recommended, even though they are categorized in class A and B for pregnancy risk.

The molecular mechanisms of resistance to antifungal antibiotics are yet to be well known for *C. albicans* strains, but the presence of merging strains is increasing in clinical practice and specific resistance patterns are developing (Table 3).

Azole resistance involves the epigenetic variety phenomenon, which is the transitory expression of certain genes related to temporary resistance or sensitivity in certain *Candida* strains. The punctiform mutations within the cytochrome P_450_-dependent 14α-sterol demethylase (P_450_DM lanosterol demethylase) enzyme inhibit azole binding but not the enzyme substrate, thus decreasing antifungal sensitivity [21]. 

The super expression of the cytochrome P_450_-dependent 14α-sterol demethylase enzyme, due to the increase in the number of copies of the target enzyme, could bring forward increases in the minimum inhibiting concentrations of antifungal drugs [22].

Biofilm formation and intracellular persistence are the characteristics of *C. glabrata* which allow it to develop antifungal resistance [23,24]. For various inflammatory and infectious diseases, drugs have an increased effect when they are based on molecules in liposomal form [25].

The complete resistance of *C. krusei* to fluconazole is demonstrative of why these mechanisms are not yet clarified [20,24]. It is assumed that the resistance to amphotericin B is developed due to mutations and the selection of resistant clones within a population, as phenotypic differences are noticed between strains that are sensitive and resistant to amphotericin. As ergosterol is the target of amphotericin B, the sterol content within the membrane is modified and structural alterations occur [24].

All six strains of *C. krusei* in our study were resistant to polyene. Particularly, the polyene resistance of *C. glabrata* is not well documented in the scientific literature, owing to the replacement of polyene-binding sterols and consecutive intracellular camouflage [23].

Resistance to 5-flucytosine is frequent and occurs mainly, in the case of monotherapy, through genetic mutations related to DNA repair, RNA, and protein metabolism [26].

Besides the specific resistance mechanisms of the antifungal class, the cross-resistance of some mechanisms was extensively developed in various antifungal agents, and sensitivity differences in antifungal drugs are expected from one strain to another [27]. The rate of infection depends on the virulence of the bacteria, the resistance factors of the host, and the regional anatomy [28]. A better understanding of the epidemiological context can be complex, but complete clinical examination and laboratory tests are important for diagnosis [29,30,31] in different hospital units because they could help clinicians to correlate the clinical manifestations of infection.

## 5. Conclusions

The present study retrospectively evaluated the sensitivity of *Candida* spp. isolated in vulvovaginitis during pregnancy. Antifungal prescriptions must account for variations in pharmacokinetics and pharmacodynamics during pregnancy. The spread of antifungal resistance limits the use of some therapeutic regimens and requires careful mycologic diagnosis. Most vaginal candidiasis in pregnancy is diagnosed in the first trimester. *C. albicans* is the main species identified in vaginal samples, while emerging species such as *C. glabrata* and *C. krusei* are also found. According to local mycologic data, topical miconazole could be recommended as a first-line antifungal treatment for vaginal candidiasis, although the complexity of resistance mechanisms in *Candida* spp. requires individual decisions to be made.

## Figures and Tables

**Table 1:** Classification of antifungal drugs by their risk in pregnancy [5].

Risk	Description	Antifungal Class	Drugs
A	The possibility of fetal harm appears remote in the first trimester because controlled studies of women have not demonstrated any risk to the fetus.	Polyenes	Nystatin
B	Either there have been no controlled studies on pregnant women and animal studies did not indicate a risk to the fetus, or animal studies have indicated fetal risks but the controlled studies did not demonstrate a risk.	Polyenes Squalene epoxidase inhibitors	Amphotericin B Terbinafine Clotrimazole
C	Either there are no available reports of studies on women or animals, or animal studies have indicated a fetal risk and there have been no controlled studies of women.	Azoles Echinocandins Antimetabolites Miscellaneous	Fluconazole low-dose regimen Itraconazole Posaconazole Caspofungin Micafungin Anidulafungin Flucytosine Griseofulvine Miconazole
D	Positive evidence of fetal risk has been reported in the literature; even so, there can be some situations in which the benefits can outweigh the risk (e.g., life-threatening or serious diseases in which other drugs are ineffective or carry a greater risk).	Azoles	Fluconazole high-dose regimen Voriconazole
X	According to studies on animals or humans, or on the basis of human experience, there is definitely a fetal risk, and the risk clearly outweighs anything that may benefit pregnant women.		

**Table 2:** Distribution of candidiasis cases depending on the sensitivity to antibiotic treatment.

	*Candida albicans*		*Candida krusei*		*Candida glabrata*	
	S	R	S	R	S	R
Amphotericin	47 (39.0%)	72 (61.0%)	0 (0%)	6 (100%)	2 (12.5%)	14 (87.5%)
Nystatin	50 (42.4%)	68 (57.6%)	0 (0%)	6 (100%)	8 (50%)	8 (50%)
Econazole	94 (79.7%)	24 (20.3%)	3 (50%)	3 (50%)	12 (75%)	4 (25%)
Fluconazole	108 (91.5%)	10 (8.5%)	2 (33.3%)	4 (66.7%)	0 (0%)	16 (100%)
Ketoconazole	100 (87.4%)	18 (15.3%)	4 (66.7%)	2 (33.3%)	10 (62.5%)	6 (37.5%)
Miconazole	110 (93.2%)	8 (6.8%)	5 (83.3%)	1 (16.7%)	14 (87.5%)	2 (12.5%)
Flucytosine	64 (54.3%)	54 (45.7%)	1 (16.7%)	5 (83.3%)	2 (12.5%)	14 (87.5%)

S = sensitive; R = resistant.

**Table 3:** The resistance mechanisms developed in *Candida* spp. [20].

Resistance Mechanism	*Candida albicans*	*Candida glabrata*	*Candida krusei*
Altered sterol composition/Erg inactivation	Yes	No	No
Exogenous sterol uptake	No	Yes	No
Increase ABC transporter expression	Yes	Yes	Yes
Increase MFS transporter expression	Yes	No	No
Low fluconazole–Erg 11 affinity	No	No	Yes
Increased Erg 11 expression	Yes	No	No
Aneuploidy	Yes	Yes	Yes
Erg11 mutations	Yes	No	No

## Data Availability

Not applicable.
